# Non-linear association of years of experience and burnout among nursing staff: a restricted cubic spline analysis

**DOI:** 10.3389/fpubh.2024.1343293

**Published:** 2024-01-31

**Authors:** Tanghong Liao, Yufei Liu, Wenqun Luo, Zhizhou Duan, Kangmin Zhan, Hongjian Lu, Xiangfan Chen

**Affiliations:** ^1^China-Australia Joint Research Center for Infectious Diseases, School of Public Health, Xi’an Jiaotong University Health Science Center, Xi’an, China; ^2^Discipline Construction Office, Jiangxi Provincial People's Hospital, The First Affiliated Hospital of Nanchang Medical College, Nanchang, China; ^3^Faculty of Health Sciences, University of Macau, Macau, China; ^4^Department of Gynecology, Jiangxi Provincial People's Hospital, The First Affiliated Hospital of Nanchang Medical College, Nanchang, China; ^5^Preventive Health Service, Jiangxi Provincial People's Hospital, The First Affiliated Hospital of Nanchang Medical College, Nanchang, China; ^6^First Clinical College, Medical College of Nanchang University, Nanchang, China; ^7^Jiangxi Medical College, Nanchang University, Nanchang, China; ^8^The Second Department of Respiratory Disease, Jiangxi Provincial People’s Hospital, The First Affiliated Hospital of Nanchang Medical College, Nantong, China; ^9^Department of Biobank, Nantong First People's Hospital, Nantong, China

**Keywords:** nurse, burn out, risk factors, year of experience, dose-response relationship

## Abstract

**Background:**

Occupational burnout is intricately linked to a spectrum of physical disorders encompassing respiratory, cardiovascular, and gastrointestinal conditions, as well as manifestations such as headaches, type 2 diabetes, elevated cholesterol levels, chronic fatigue, and muscle pain. Despite this association, there remains a paucity of research on the specific risk factors contributing to burnout among nurses in China.

**Methods:**

This cross-sectional study utilized convenience sampling to recruit participants, with data analyzed from 1,774 nurse staffs. Psychosocial traits were assessed using the Three-Item Loneliness Scale for loneliness, the Generalized Anxiety Disorder 7-item scale (GAD-7) for anxiety symptoms, the 9-item Patient Health Questionnaire (PHQ-9) for depressive symptoms, the Connor Davidson Resilience Scale–10 item (CDRISC-10) for resilience, and the Maslach Burnout Inventory-Human Service Survey (MBI-HSS) for burn out. Restrictive cubic spline analysis to investigate the dose-response relationship between years of experience and burn out. Multivariate linear regression was employed to investigate the relationship between burnout and various risk factors.

**Results:**

After controlling for basic demographic variables, good sleep quality was associated with a reduction in emotional exhaustion (β = −0.307, *p* < 0.001), while loneliness (β = 1.334, *p* < 0.001), depressive symptoms (β = 0.896, *p* < 0.001), and anxiety symptoms (β = 0.504, *p* < 0.001) were significantly associated with increased emotional exhaustion. Moreover, higher levels of resilience were positively associated with personal accomplishment (β = 0.635, *p* < 0.001). Regarding depersonalization, loneliness (β = 0.577, *p* < 0.001), depressive symptoms (β = 0.429, *p* < 0.001), and anxiety symptoms (β = 0.152, *p* < 0.01) were found to increase its level. Conversely, good resilience was associated with a decrease in depersonalization (β = −0.069, *p* < 0.001). The non-liner association between year of experience and emotional exhaustion was significantly (*p* < 0.05).

**Conclusion:**

Our findings revealed that significant risk factors contributing to burnout among nursing staff including bad sleep quality, loneliness, lower level of resilience, anxiety symptoms, depressive symptoms. Moreover, a nonlinear correlation between years of experience and the likelihood of experiencing emotional exhaustion was exist.

## Introduction

The well-being of nurses is absolutely vital to the operation of the healthcare system and the quality of patient care. Roughly 40% of nurses encounter burnout, which is the most extensively researched indicator of less-than-ideal well-being (1, 2). This phenomenon encompasses three core elements: emotional exhaustion (EE), characterized by a gradual depletion of energy, resulting in fatigue, weariness, and physical or psychological manifestations, or a combination of both; depersonalization (DP), involving negative behaviors and indifferent responses toward those under care or treatment; and low personal accomplishment (PA), marked by a sense of limitation and a lack of fulfillment in the work performed (3). The extensive impact of clinician burnout on the health system has been extensively studied. Dissatisfaction with jobs and the intention to change positions or professions have been linked to poor well-being (4). Organizations experience significant costs as a result of nurse turnover, amounting to approximately 1.2 times the annual salary of each replaced nurse (5, 6). The likelihood of nurses making medical errors increases by 26–71% when they experience suboptimal well-being (7). Additionally, burnout is associated with various physical disorders including respiratory, heart, and gastrointestinal conditions, headaches, type 2 diabetes, high cholesterol levels, chronic fatigue, and muscle pain (8). Hence, it is crucial to address nurses’ burnout in order to guarantee economical, safe, and effective patient care.

To effectively intervene in nurse burnout, it is necessary and crucial to understand its influencing factors. Several factors may contribute to nurse burnout, including workload, moral distress, a flawed support system, limited resources, limited training, and bullying (9). Additionally, anxiety and depression may result from prolonged exposure to stress, leading to nurse burnout (9). Furthermore, demographic variables such as gender and education level are also important factors in nurse burnout (1, 10). Despite the known contributing factors, there are still some psychological factors that have not been adequately documented, such as sleep quality and loneliness. Therefore, this research will further investigate the issue, including resilience in China.

Years of experience plays a vital role in influencing nurse burnout. In Sulaiman’s research, multiple logistic regression analysis uncovers that the willingness of nurses to assume leadership roles is associated with a specific demographic factor, specifically, the number of years they have worked in the profession (11). This discovery aligns with the findings of Bulmer (12), who observed that nurses’ eagerness to take on leadership positions tends to diminish as they accumulate more experience over time. Senior nurses with extensive experience often exhibit less interest in leadership roles compared to their junior counterparts with less than 2 years of experience. This decline in leadership motivation can be attributed to the cumulative impact of long-term careers in the healthcare field (12). As nurses advance in their careers, they encounter heightened work-related stress and professional fatigue, leading to both physical and emotional exhaustion. This is primarily a result of their dual responsibilities, encompassing the care of patients’ medical needs and navigating intricate healthcare scenarios and workplace challenges. In the context of precise interventions to address nurse burnout, it is crucial to consider the impact of years of work experience. However, only a limited number of studies have delved into the dose-response relationship and the interplay between years of experience and burnout in nursing staff.

In this study, our main goals were to initially assess burnout risk factors, which include sleep quality, loneliness, and resilience. Subsequently, we employed restrictive cubic spline analysis to investigate the dose-response relationship and the interplay between years of experience and burnout among nursing staff.

## Methods

### Participants and procedures

In the Dehong districts of Yunnan province, a comprehensive cross-sectional survey was conducted. Invitations to participate in this research were extended to the nursing staff from all 18 government-operated hospitals in the Dehong districts. The participant pool was assembled using a convenience sampling strategy. The participants were provided with the ability to respond to our questionnaires through Wenjuanxing, China’s most extensive online survey platform. The nursing departments of each government hospital played a crucial role in disseminating the link to our questionnaire. The survey was designed to ensure participant anonymity, and we encouraged participants to complete the questionnaire independently. Participants were given the freedom to contact our researchers with any questions or concerns about the survey. To be eligible for inclusion in this study, participants needed to meet the following conditions: (1) They were currently employed at one of the 18 local government hospitals; (2) They were capable of comprehending the content of the questionnaire; (3) They demonstrated a willingness to participate and provided informed consent; (4) They did not have any diagnosed mental illness; (5) They were not student nurses. We made sure to inform the nursing staff about their rights, including the option to withdraw from the survey at any stage. The questionnaire completion process took approximately 7 min for most participants. In total, we extended invitations to 1,965 nursing staff to participate in our research. Of these, 1,774 completed the survey, resulting in a response rate of 90.3%. The Ethics Committee of Dehong People’s Hospital in China (Number: DYLL-KY032) provided approval for this research.

### Measures

#### Socio-demographic variables

The basic socio-demographic characteristics of the participants were included the number of years they had been working, their gender, ethnicity, place of residence, level of education, and whether they were an only child.

#### Sleep quality

The quality of sleep among the participants was evaluated using the Single-Item Sleep Quality Scale (SQS) (13). Participants were asked to respond to the question, “During the past 7 days, how would you rate your sleep quality overall?” They were provided with an 11-point scale for responses, which ranged from 0 (indicating terrible sleep quality) to 10 (indicating excellent sleep quality). Higher scores on this scale denoted better sleep quality.

#### Loneliness

Loneliness among the participants was measured using the Three-Item Loneliness Scale (14). This tool employs a 3-point Likert scale with responses ranging from “hardly ever,” “some of the time,” to “often.” An example of an item from the scale is, “How often do you feel that you lack companionship?” The total score was calculated by summing the scores of each item, with possible scores ranging from 3 to 9. Higher cumulative scores indicated a more severe level of loneliness. In this study, the Cronbach’s alpha for this scale was 0.80.

#### Depressive symptoms

Depressive symptoms among the participants were gaged using the 9-item Patient Health Questionnaire (PHQ-9) (15). This tool incorporates a 4-point Likert scale with responses ranging from “not at all,” “several days,” “more than half the days,” to “nearly every day,” with corresponding scores from 0 to 3. The total score for this scale could range from 0 to 27, with higher scores indicating more severe depressive symptoms. The Chinese version of the PHQ-9 has demonstrated good validity and reliability within the Chinese context. In this study, the Cronbach’s alpha for the PHQ-9 was 0.91.

#### Anxiety symptoms

Anxiety symptoms among the participants were assessed using the Generalized Anxiety Disorder-7 (GAD-7) scale (16). This tool consists of 7 items and employs a 4-point response scale, ranging from 0 (indicating “not at all”) to 3 (indicating “nearly every day”). The total score for this scale could range from 0 to 21, with higher cumulative scores indicating more severe anxiety symptoms. The Chinese version of the GAD-7 has shown good validity and reliability within the Chinese context. In this study, the Cronbach’s alpha for the GAD-7 was 0.9.

#### Resilience

Resilience among the participants was evaluated using the Connor Davidson Resilience Scale – 10 item (CDRISC-10) (17). This tool comprises 10 items and utilizes a 5-point response scale, ranging from 0 (indicating “never true”) to 4 (indicating “always true”). The total score for this scale was calculated by summing the scores of each item, with higher scores indicating stronger resilience levels. The CDRISC-10 has shown good validity and reliability within the Chinese context (18). In this study, the Cronbach’s alpha for the CDRISC-10 was 0.94.

#### Occupational burnout

The Chinese version of the Maslach Burnout Inventory-Human Service Survey (MBI-HSS) (19, 20), which comprises three subscales, was used to measure occupational burnout among the nurses. The three subscales include emotional exhaustion (9 items, e.g., “I feel emotionally drained from my work,” with a Cronbach’s alpha of 0.92), personal accomplishment (8 items, e.g., “I have accomplished many worthwhile things in this job,” with a Cronbach’s alpha of 0.83), and depersonalization (5 items, e.g., “I feel I treat some recipients as if they were impersonal objects,” with a Cronbach’s alpha of 0.87). Each item is evaluated using a seven-point Likert scale that ranges from 0 (indicating “not at all”) to 6 (indicating ‘nearly every day’). The scores for the items in each subscale are summed to obtain the total score for each dimension. This comprehensive approach allows for a detailed assessment of the different facets of occupational burnout.

### Statistical analysis

#### Descriptive analysis

For the qualitative data, the frequency and percentage (N/%) were used to present the data. On the other hand, for quantitative data, we conducted a normality test on the quantitative data using the Shapiro–Wilk method. Upon confirmation of normality, we utilized the mean ± standard deviation (SD) for description. In cases where the data did not adhere to normality, we opted to describe using quartile percentages.

#### Multivariate liner regression

In the study, multivariate linear regression model was employed to examine the effects of work years and psychosocial variables on burn out. Multivariate liner regression model also controlled basic socio-demographic covariates.

#### Dose-response analysis

We conducted a non-linear analysis to investigate the relationship between work years and burnout using restricted cubic splines (RCS). In this study, the data were adjusted for several covariates including sex, ethnicity, residence, education level, only children, sleep quality, loneliness, depressive symptoms, anxiety symptoms, and resilience. The linear regression model was fitted to the data, and the RCS technique was applied with 5 knots placed at the 5th, 27.5th, 50th, 72.5th, and 95th percentiles of work years. The reference point for comparison was set at the 5th percentile. To assess the significance of non-linear trends, Wald tests were conducted for the RCS coefficients. These tests provide *p*-values that indicate whether the non-linear relationship between work years and burnout is statistically significant.

RCS analysis was performed using R version 3.6.2, while other statistical analyses were conducted using SPSS version 22.0. The significance level was set at 0.05 for all statistical tests, and a two-tailed approach was used.

## Results

A total of 1,774 nurses were included in the statistical analysis. The baseline socio-demographic characteristics and psychological outcomes of the participants are presented in Table 1. Among the nurses, the majority were female (1,666, 93.9%), of Han ethnicity (1,276, 71.9%), only children (1,491, 84.0%), residing in rural areas (1,071, 60.4%), and holding a Bachelor’s degree or above (1,160, 65.4%). Because it does not meet the normality test, the measurement variable is described using quartiles: The median of work experience of the nurses was 8.00 years (5.00, 14.00). In terms of mental health outcomes, the mean score for sleep quality was 6.00 (5.00, 8.00), loneliness was 3.00 (1.00, 3.00), anxiety symptoms were 6.00 (3.00, 8.00), depressive symptoms were 7.00 (4.00, 10.00), and resilience was 21.00 (17.00, 28.00). Furthermore, the MBI-HSS scores indicated that the median emotional exhaustion was 18.00 (10.00, 28.00), personal accomplishment was 29.00 (21.00, 38.00), and depersonalization was 5.00 (1.00, 11.00) among the nurses.

**Table 1:** Socio-demographic and psychological characteristics of the study participants (*N* = 1774).

Characteristic	Number	Percent (%)
**Sex**		
Women	1,666	93.9
Men	108	6.1
**Ethnic**		
Han	1,276	71.9
Others	498	28.1
**Residence**		
Rural	1,071	60.4
Urban	703	39.6
**Education level**		
High school or lower	614	34.6
Bachelor’s degree or above	1,160	65.4
**Only child**		
Yes	283	16.0
No	1,491	84.0
**Key variables (Mean ± SD/25, 50, 75%)**		
Sleep quality	6.00 (5.00, 8.00)	
Loneliness	3.00 (1.00, 3.00)	
Depressive symptoms	7.00 (4.00, 10.00)	
Anxiety symptoms	6.00 (3.00, 8.00)	
Resilience	21.00 (17.00, 28.00)	
Work years	8.00 (5.00, 14.00)	
MBI-HSS emotional exhaustion	18.00 (10.00, 28.00)	
MBI-HSS personal accomplishment	29.00 (21.00, 38.00)	
MBI-HSS depersonalization	5.00 (1.00, 11.00)	

**Figure 1:**
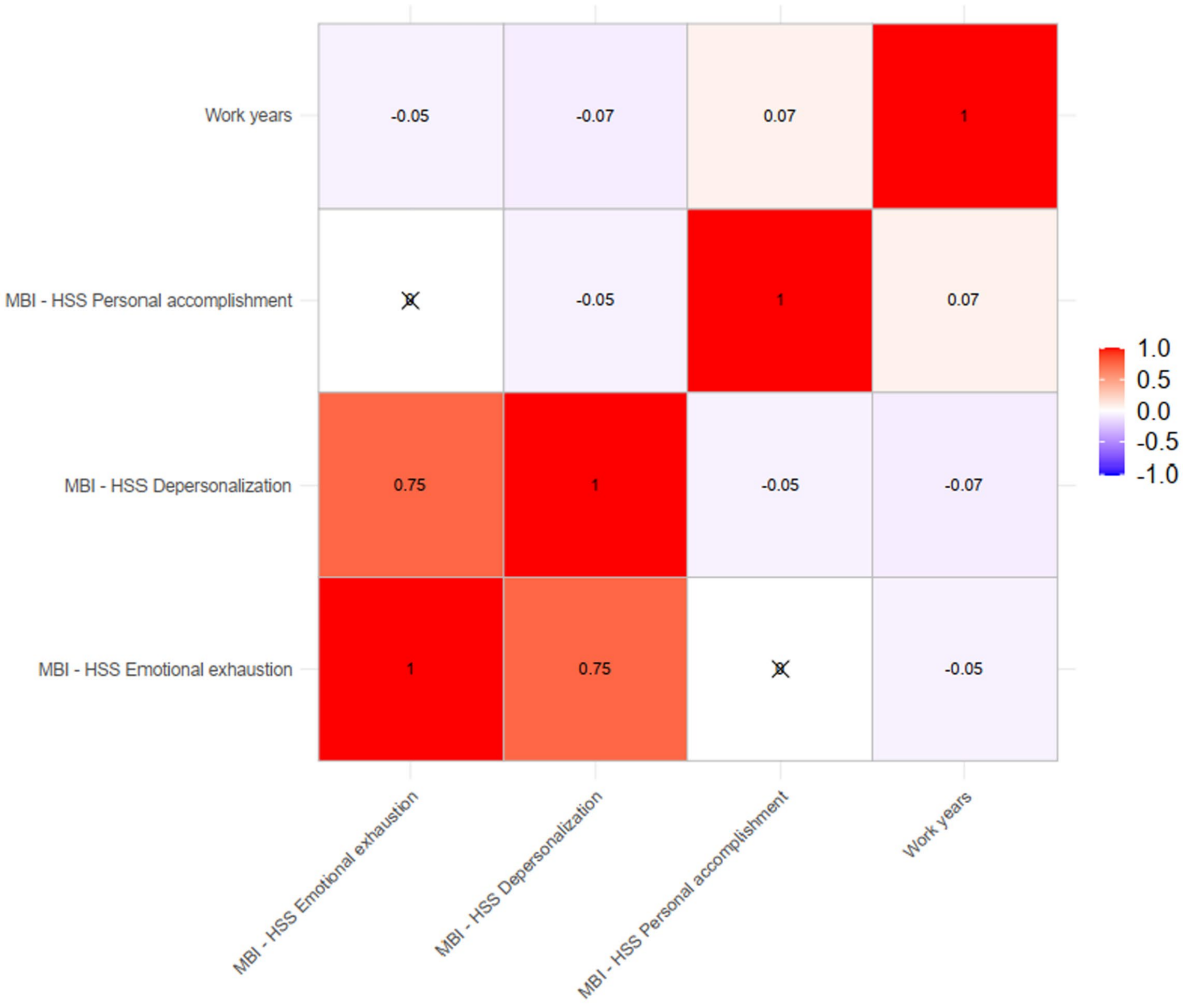
Correlation analysis of the association between work years and burn out.

As presented in the Figure 1, we conducted a correlation analysis to investigate the association between work years and burnout. The results indicated a significant correlation between work years and burnout, with a correlation coefficient of −0.05 (*p* < 0.05) for emotional exhaustion, 0.09 (*p* < 0.01) for personal accomplishment, and −0.07 (*p* < 0.01) for depersonalization.

Table 2 displays the results of linear regression analyses examining the associations between work years, burnout, and psychological characteristics. In all three models, an increase in work years was found to significantly decrease nurses’ emotional exhaustion (β = −0.102, *p* < 0.001) and depersonalization (β = −0.058, *p* < 0.001). However, the association between work years and personal accomplishment was not significant in model 3 (β = 0.003, *p* > 0.05), although it was significant in model 1 (β = 0.093, *p* < 0.01). After controlling for basic demographic variables, additional findings revealed that good sleep quality was associated with a reduction in emotional exhaustion (β = −0.307, *p* < 0.001), while loneliness (β = 1.334, *p* < 0.001), depressive symptoms (β = 0.896, *p* < 0.001), and anxiety symptoms (β = 0.504, *p* < 0.001) were significantly associated with increased emotional exhaustion. Moreover, higher levels of resilience were positively associated with personal accomplishment (β = 0.635, *p* < 0.001). Regarding depersonalization, loneliness (β = 0.577, *p* < 0.001), depressive symptoms (β = 0.429, *p* < 0.001), and anxiety symptoms (β = 0.152, *p* < 0.01) were found to increase its level. Conversely, good resilience was associated with a decrease in depersonalization (β = −0.069, *p* < 0.001).

**Table 2:** Liner regression analysis of the association between work year and burn out.

Variables	MBI-HSS emotional exhaustion			MBI-HSS personal accomplishment			MBI-HSS depersonalization		
	Model1	Model 2	Model 3	Model1	Model 2	Model 3	Model1	Model 2	Model 3
Work years	−0.077*	−0.118**	−0.102***	0.093**	0.066	0.033	−0.055***	−0.066***	−0.058***
Sex (female)		2.724*	1.001		−0.074	1.234		0.239	−0.609
Ethnic (others)		0.874	−0.177		−2.277***	−1.764***		0.952**	0.470
Residence (urban)		0.833	0.732		1.454*	1.234*		0.169	0.149
Education level (Bachelor’s degree or above)		0.676*	0.457		0.182	−0.026		0.202	0.130
Only child (yes)		−0.469	0.097		1.851*	1.736*		−0.283	0.029
Sleep quality			−0.307***			0.101			−0.030
Loneliness			1.334***			0.001			0.557***
Depressive symptoms			0.896***			0.090			0.429***
Anxiety symptoms			0.504***			−0.116			0.152**
Resilience			−0.039			0.635***			−0.069***

*p < 0.05; **p < 0.01; ***p < 0.001.

**Figure 2:**
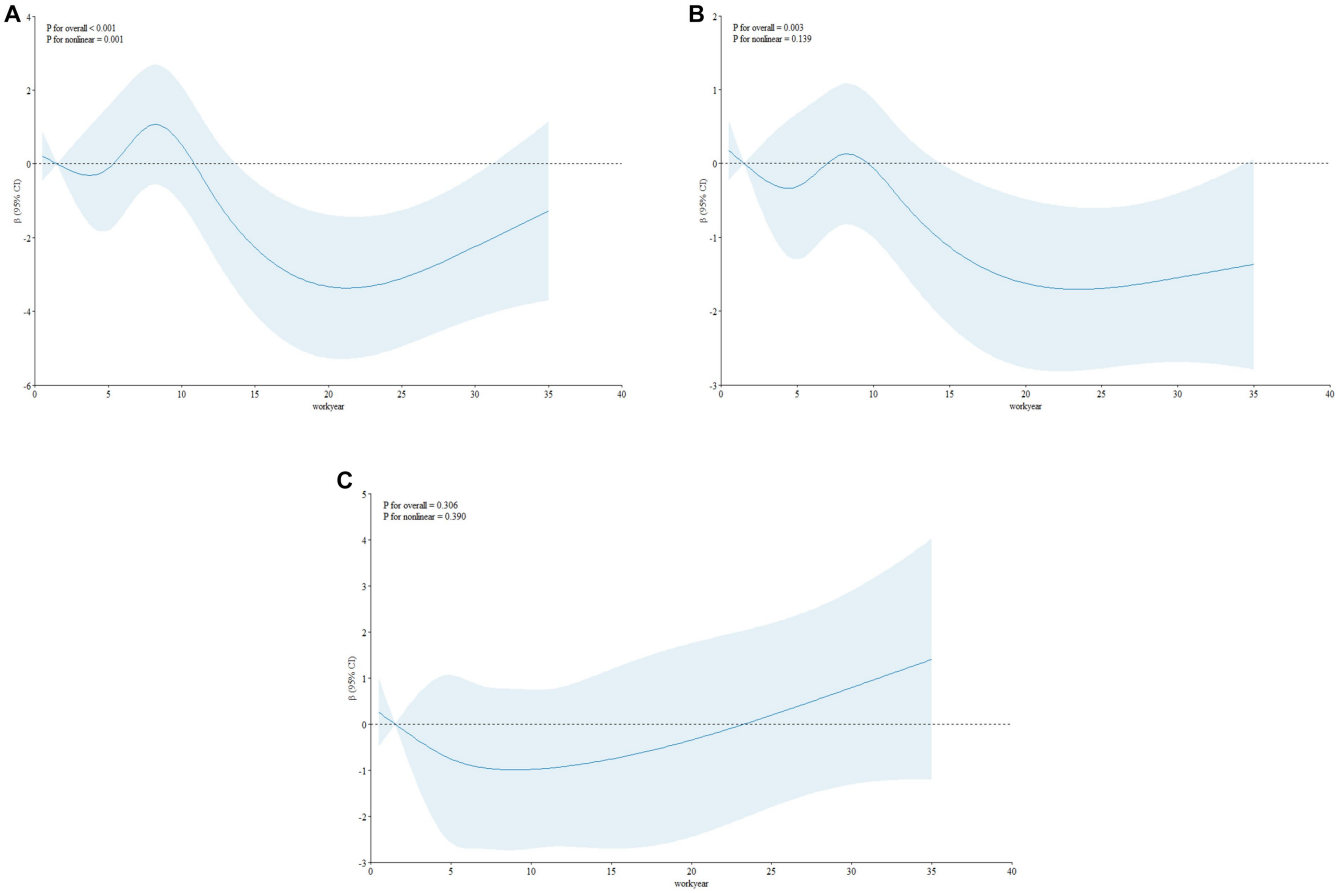
**(A)** Association Between work year and MBI-HSS Emotional exhaustion using a Restricted Cubic Spline Regression Model. Graphs show β for MBI-HSS Emotional exhaustion according to work year adjusted for sex, ethnic, residence, education level, only children, sleep quality, loneliness, depressive symptoms, anxiety symptoms, resilience. Data were fitted by a linear regression model, and the model was conducted with 5 knots at the 5th, 27.5th, 50th, 72.5th, 95th percentiles of work year (reference is the 5th percentile). Solid lines indicate β, and shadow shape indicate 95% CIs. CI, confidence interval. **(B)** Association Between work year and MBI-HSS Depersonalization using a Restricted Cubic Spline Regression Model. Graphs show β for MBI-HSS Emotional exhaustion according to work year adjusted for sex, ethnic, residence, education level, only children, sleep quality, loneliness, depressive symptoms, anxiety symptoms, resilience. Data were fitted by a linear regression model, and the model was conducted with 5 knots at the 5th, 27.5th, 50th, 72.5th, 95th percentiles of work year (reference is the 5th percentile). Solid lines indicate β, and shadow shape indicate 95% CIs. CI, confidence interval. **(C)** Association Between work year and MBI-HSS Personal accomplishment using a Restricted Cubic Spline Regression Model. Graphs show β for MBI-HSS Emotional exhaustion according to work year adjusted for sex, ethnic, residence, education level, only children, sleep quality, loneliness, depressive symptoms, anxiety symptoms, resilience. Data were fitted by a linear regression model, and the model was conducted with 5 knots at the 5th, 27.5th, 50th, 72.5th, 95th percentiles of work year (reference is the 5th percentile). Solid lines indicate β, and shadow shape indicate 95% CIs. CI, confidence interval.

Multivariable adjusted restricted cubic spline analyses were conducted to examine the nonlinear relationships between work years and burnout (Figures 2A–C). The models were adjusted for sex, ethnicity, residence, education level, being an only child, sleep quality, loneliness, depressive symptoms, anxiety symptoms, and resilience. Figure 2A displays the relationship between work years and emotional exhaustion, showing a significant nonlinear association (*p* values for non-linearity <0.001). Initially, as work years increased, emotional exhaustion levels decreased. However, at around 4 work years, emotional exhaustion began to increase and reached its peak at approximately 8 years. Subsequently, with further increases in work years, emotional exhaustion continued to decline until around 21 years, after which it started to increase again, albeit with a reduced magnitude. In Figure 2B, the overall association between work years and depersonalization was found to be statistically significant (*p* = 0.003). However, the specific non-linear association between work years and depersonalization was not significant (*p* = 0.139), following a similar trend as emotional exhaustion. Regarding personal accomplishment, there were no significant overall associations (*p* = 0.306) or nonlinear relationships (*p* = 0.390) observed between work years and personal accomplishment.

## Discussion

In this cross-sectional study examining nurse staff, several significant findings emerged. Good sleep quality was associated with a reduction in emotional exhaustion, while loneliness was linked to increased emotional exhaustion and depersonalization. Higher levels of resilience were positively related to personal accomplishment and a decrease in depersonalization. Additionally, the study revealed a non-linear relationship between years of experience and the risk of emotional exhaustion among nurse staff. These findings highlight the intricate connections between sleep quality, loneliness, resilience, years of experience, and various dimensions of burnout in nursing professionals.

Our study show that sleep quality has been linked to lowered emotional exhaustion. Researchers across various global regions have conducted multiple studies to understand the connection between sleep quality and burnout. For example, a study involving shift-work nurses in Italy found evidence supporting a link between poorer sleep quality and heightened burnout (21). Similarly, Brazilian nursing personnel who expressed dissatisfaction with their sleep were more prone to experiencing Emotional Exhaustion (22). A restful night’s sleep appears to enhance our well-being, enabling us to better navigate the emotional challenges of the following day, especially when confronted with emotionally distressing events. Sleep seems to play a restorative role in our daily functioning, while sleep deprivation makes us notably more susceptible to emotional and stressful stimuli and events (23). Thus, nurse staff workers who obtain sufficient and good-quality sleep are more equipped to handle the emotional demands of their daily work. Conversely, those experiencing poor sleep quality are at a higher risk of susceptibility to stress and burnout.

Our study also revealed a correlation between loneliness and increased emotional exhaustion and depersonalization, consistent with prior research (24). Similarly, existing literature within comparable populations supports the notion that loneliness serves as a predictor of burnout, with estimates suggesting that loneliness can explain approximately one third of the variation in burnout levels (24). Studies involving medical residents have highlighted a link between loneliness and burnout, emphasizing the role of robust social networks in reducing the incidence of burnout (25, 26). Resilience is a crucial factor contributing to burnout. Higher levels of resilience have been found to be positively associated with personal accomplishment and a reduction in depersonalization (27). Enhanced resilience has also been linked to an improvement in nurse mental health and a greater willingness to work effectively (27). The findings regarding the relationships between resilience and the three dimensions of burnout in this study align with existing empirical evidence, which indicates that resilience is linked to lower levels of burnout and acts as a preventive factor against burnout syndrome in critical care professionals (28, 29).

In addition, our study revealed that nurses who experience heightened levels of anxiety and depression often demonstrate increased rates of burnout stemming from the demanding and adverse work environment. Consequently, this contributes to a deterioration in their overall quality of life, accompanied by a waning interest and escalating frustration, potentially culminating in their departure from the profession (30). As such, it is imperative to introduce interventions focused on enhancing mental well-being and fostering effective coping strategies (31, 32).

Our study revealed a non-linear relationship between years of experience and the risk of emotional exhaustion among nurse staff. Previous study have shown that years of experience was an important factors of burn out in nurse staff. In a study conducted by Kelly at a prominent quaternary care teaching facility in the southwestern United States, she and her colleagues unveiled that increased years of experience among nurses significantly predicted burnout (33). Simultaneously, Carlos and his colleagues, through a two-population comparative pilot study, revealed that more years of experience exert a greater influence on the increase in emotional exhaustion and depersonalization. Specifically, individuals with over 5 years of experience exhibited elevated values in these aspects (34). A survey conducted among Turkish dentists revealed that after 15–20 years of experience, they reported increased emotional exhaustion and reduced personal fulfillment (35). In this study, we not only discovered the relationship between years of work experience and burnout but also utilized the RCS method to delve deeper into the non-linear connection between years of experience and emotional exhaustion among nursing staff. Our findings revealed an initial decrease in emotional exhaustion levels as work years increased. However, at approximately 4 years of work experience, emotional exhaustion began to rise, reaching its peak at around 8 years. Subsequently, with further increases in work years, emotional exhaustion continued to decline until reaching a minimum at around 21 years. For future interventions targeting burnout in nurses, hospital management should pay particular attention to the critical period around 8 years of work experience. This underscores the importance of addressing burnout concerns among nursing staff, especially those with 8 years of experience.

This study is subject to several limitations that warrant consideration. Firstly, the cross-sectional design constrains the extrapolation of results and the ability to infer causality. To gain a more comprehensive understanding of the causal relationships between years of experience and staff burnout, further longitudinal research is imperative. Secondly, participants were recruited through convenient sampling from a single, specific location in China, potentially restricting the generalizability of findings to a broader, nationally representative sample. Thirdly, inherent reporting and recall biases may persist in this study, emphasizing the need for caution in interpreting the results.

## Conclusion

The study uncovered significant risk factors contributing to burnout among nursing staff while also exploring the intricate relationship between years of experience and burnout levels. Notably, sleep quality, loneliness, and resilience emerged as crucial elements influencing burnout. Specifically, adequate sleep quality was correlated with a decrease in emotional exhaustion. Conversely, feelings of loneliness were associated with heightened emotional exhaustion and depersonalization. Furthermore, higher resilience levels were linked to increased personal accomplishment and a reduction in depersonalization. Moreover, the research highlighted a nonlinear correlation between years of experience and the likelihood of experiencing emotional exhaustion among nursing staff.

## Data availability statement

The data analyzed in this study is subject to the following licenses/restrictions: Data available on request from authors. Requests to access these datasets should be directed to ZD, 2013302170005@whu.edu.cn.

## Ethics statement

The studies involving humans were approved by The Ethics Committee of Dehong People’s Hospital in China (Number: DYLL-KY032). The studies were conducted in accordance with the local legislation and institutional requirements. Written informed consent for participation in this study was provided by the participants' legal guardians/next of kin.

## Author contributions

TL: Conceptualization, Formal analysis, Funding acquisition, Supervision, Writing – original draft. ZD: Conceptualization, Funding acquisition, Investigation, Supervision, Writing – original draft. KZ: Conceptualization, Funding acquisition, Supervision, Writing – original draft. HL: Formal analysis, Writing – original draft, Writing – review & editing. XC: Formal analysis, Writing – original draft, Writing – review & editing. YL: Writing - review & editing. WL: Writing - review & editing.
